# Naringin attenuates cisplatin‐ and aminoglycoside‐induced hair cell injury in the zebrafish lateral line via multiple pathways

**DOI:** 10.1111/jcmm.16158

**Published:** 2020-12-03

**Authors:** Ming Li, Jingwen Liu, Dong Liu, Xuchu Duan, Qingchen Zhang, Dawei Wang, Qingyin Zheng, Xiaohui Bai, Zhiming Lu

**Affiliations:** ^1^ Department of Clinical Laboratory Shandong Provincial Hospital Cheeloo College of Medicine Shandong University Jinan China; ^2^ Department of Orthopaedics Shandong Provincial Hospital Cheeloo College of Medicine Shandong University Jinan China; ^3^ College of Life Science Nantong University Nantong China; ^4^ Department of Otolaryngology‐Head & Neck Surgery Case Western Reserve University Cleveland OH USA

**Keywords:** aminoglycosides, cisplatin, naringin, ototoxicity, therapeutic

## Abstract

Exposure to ototoxic drugs is a significant cause of hearing loss that affects about 30 thousand children with potentially serious physical, social and psychological dysfunctions every year. Cisplatin (CP) and aminoglycosides are effective antineoplastic or bactericidal drugs, and their application has a high probability of ototoxicity which results from the death of hair cells (HCs). Here, we describe the therapeutic effect of the flavonoid compound naringin (Nar) against ototoxic effects of cisplatin and aminoglycosides include gentamicin (GM) and neomycin (Neo) in zebrafish HCs. Animals incubated with Nar (100‐400 μmol/L) were protected against the pernicious effects of CP (150‐250 μmol/L), GM (50‐150 μmol/L) and Neo (50‐150 μmol/L). We also provide evidence for the potential mechanism of Nar against ototoxicity, including antioxidation, anti‐apoptosis, promoting proliferation and hair cell regeneration. We found that mRNA levels of the apoptotic‐ and pyroptosis‐related genes are regulated by Nar both in vivo and in vitro. Finally, by proving that Nar does not affect the anti‐tumour efficacy of CP and antibacterial activity of aminoglycosides in vitro, we highlight its value in clinical application. In conclusion, these results unravel a novel therapeutic role for Nar as an otoprotective drug against the adverse effects of CP and aminoglycosides.

## 1 INTRODUCTION

Ototoxicity refers to the pathological and functional impairments of auditory nervous system caused by the application of some therapeutic drugs. Although drug‐induced hearing loss is not fatal, it will seriously damage the social and health‐related quality of patients, with significant implications for careers, education and society. In children, even minor hearing loss can impede speech, language, cognition and social development, which can lead to poor academic performance and psychosocial functioning.^1^, ^2^, ^3^, ^4^, ^5^ Worldwide, around 30 thousand children suffer from cytotoxic agents every year. Currently, there are more than 600 drugs having ototoxic properties, such as aminoglycoside antibiotics, platinum‐based chemotherapy drugs, cyclic diuretics, macrolides and antimalarials. These drugs are fully effective against a variety of infections and malignancies in children and adults.^6^ A recent interview‐based report showed that doctors tended to consider alternative medicines if ototoxicity is detected in patients.^7^ However, ototoxic drugs are still widely used in most developing countries, due to economic conditions and no prescription‐restriction.^8^ Therefore, reducing the ototoxicity of clinical drugs while ensuring their therapeutic efficacy is a problem that needs to be solved at present.

Currently, multiple studies have been performed to screen otoprotective compounds that can alleviate drug‐mediated ototoxicity, and many compounds have achieved excellent results in animal and in vitro experiments.^9^, ^10^, ^11^, ^12^ Unfortunately, although several clinical trials are underway, there are no drugs approved by the US Food and Drug Administration for the prevention and treatment of ototoxicity. The main reason for this is that the mechanisms by which these commonly used drugs cause deafness involve multiple factors and steps that cause different patients to respond differently to the same treatment. Barrel Theory may explain this: weaknesses are the limiting factor to success. Therefore, the ideal prevention and control measures should be able to play a role in many aspects.

Cisplatin (CP) is an initial therapy for malignant tumours, such as head and neck tumours (nasopharyngeal cancer, etc), which causes severe ototoxic side‐effects. CP exerts its anti‐tumour activity by forming crosslinks with DNA to disrupt its structure. CP‐DNA adducts can bind to other proteins, inhibit DNA repair, and further activate cell signal transduction pathways, resulting in cell cycle arrest and apoptosis.^13^ Many previous studies have shown that the ototoxicity of CP is mainly caused by inducing sensory hair cells (HCs) death in the inner ear. Increasing evidence report that CP can enter the cytoplasm of HCs via stereociliary mechanoelectrical transduction (MET) channels, inducing mitochondrial DNA damage, and the accumulation of reactive oxygen species.^14^

Aminoglycosides are a highly effective antibiotic, such as neomycin (Neo) and gentamicin (GM). Aminoglycosides function by binding to bacterial ribosomal 30 s subunits to induce mRNA mistranslation or premature termination, thus inhibiting bacterial reproduction.^15^ Pathological studies have found that the ototoxicity caused by aminoglycosides includes the cochlea and vestibule of the inner ear, and HCs are considered as the main damage targets.^16^ Aminoglycosides enter the outer HCs through the MET channels, forming an electron donor complex with iron, such as arachidonic acid, which produces reactive oxygen species. Reactive oxygen species can activate the c‐Jun N terminal kinase (JNK) pathway and then transfer to the nucleus to activate the transcription of genes in cell death pathway. These genes products are then transferred to the mitochondria, inducing the release of cytochrome c and causing cell apoptosis.^17^

In developed countries, complementary or alternative medicine (CAM) is increasingly used to treat a variety of diseases. Considering the lack of reliable otoprotective drug agents in modern medicine, plant‐derived phytoconstituents seem to be a noteworthy choice. Dietary flavonoids are rich in many plants and are considered to be the most effective plant antioxidants in vivo and vitro.^18^ Naringin (Nar), as a member of the flavonoid family, has a variety of pharmacological effects, such as antioxidant, cholesterol‐lowering, anti‐cancer, antibacterial, anti‐apoptotic, metal chelation and antimutagenic activities.^19^ There is evidence that Nar can antagonize CP‐induced hepatotoxicity by reducing the levels of glutamic pyruvic transaminase, glutamic oxaloacetic transaminase, alkaline phosphatase and malondialdehyde and increasing the levels of glutathione, superoxide dismutase and catalase.^20^ This suggests Nar has the potential to be an ideal measure for the prevention and treatment of drug‐mediated ototoxicity. However, no studies have investigated the effects of Nar on HCs, especially in cell proliferation, development and ototoxicity resistance.

**FIGURE 1:**
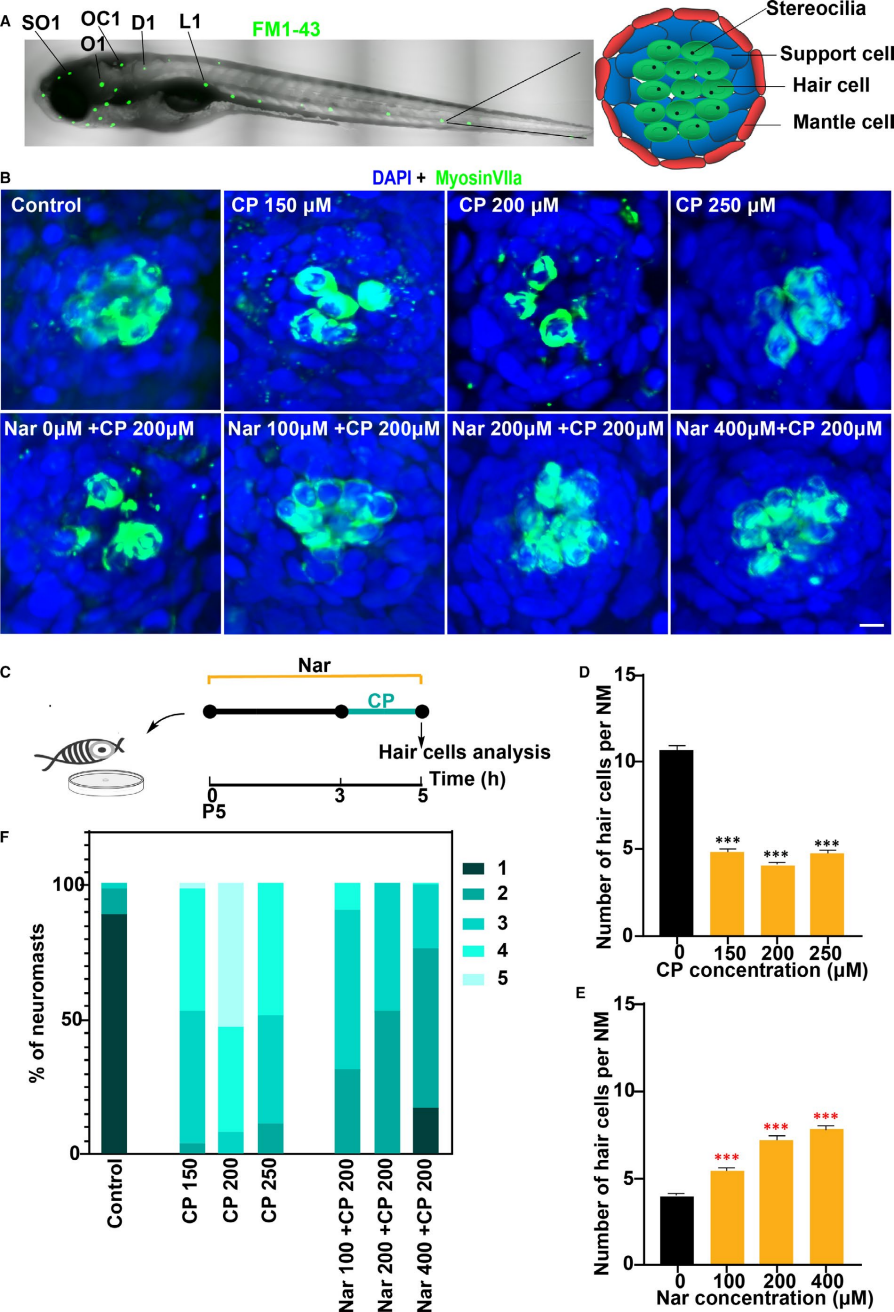
Nar protects against cisplatin ototoxicity. (A) Confocal image of a 5 d post‐fertilization (dpf) zebrafish. The neuromasts analysed in this experiment are indicated by black lines: SO1(supraorbital 1), O1 (otic 1), OC1(occipital 1), D1(dorsal trunk 1) and L1(lateral 1). Schematic top view of a 5 dpf neuromast showing the different cell types is shown in the right. (B) Animals were fixed and immunostained for MyosinVIIa (green) and DAPI. 5dpf zebrafish were incubated with 0, 150, 200 and 250 μmol/L cisplatin (CP) for 2 h, or pre‐treated with 0 µmol/L to 400 µmol/L of Naringin(Nar) for 3 h and then co‐treated with Nar and CP (150 µmol/L) for 2 h. (C) Diagram of the assay for B. (D and E) Quantification of the number of hair cells (MyosinVIIa^+^) per neuromast after the different treatments represented as mean ± SEM (n = 10). Control animals were exposed to vehicle alone (DMSO). ***P < 0.001. Black asterisks compared to DMSO‐treated animals. Red asterisks compared versus the corresponding CP concentration. (F) Scores for neuromast morphology of each group (The grading criteria are shown in the Materials and Methods). Scale bar in A equals 500 μm, B equals 5 μm

The zebrafish lateral line system has emerged as an excellent animal model to study the function of human HCs.^21^ The sensory organs (neuromasts) of zebrafish lateral line system structurally consist of mantle cells on the outside and support cells and mechanosensory HCs in the centre (Figure 1A). The hair cell‐rich neuromast of zebrafish is located on the lateral line of the ear sac and body surface, which is convenient for living staining and observation. Zebrafish HCs are structurally and functionally similar to those in mammals and also affected by the same ototoxic drugs including CP and aminoglycosides.^22^ Therefore, zebrafish can be used as a suitable model for the study of hair cell protective agent.^23^ In this study, we found that Nar could alleviate the damage of zebrafish lateral line HCs caused by CP and aminoglycosides. In terms of mechanism, we proved that Nar could decrease the intracellular accumulation of reactive oxygen species induced by ototoxic drugs, reduce cells apoptotic and promote cell proliferation in the neuromast. In addition, we also found that Nar was involved in the repair of zebrafish HCs by promoting cell regeneration.

Our study supports the new role of Nar in the protection of HCs in the lateral line of zebrafish and emphasizes its potential application in the treatment of drug‐induced hearing loss.

## 2 EXPERIMENTAL SECTION

### 2.1 Animals

The zebrafish used in this study were gifted by Dr Liu Dong from Nantong University (Nantong, China). The animal experimental protocol was approved by the Animal Ethics Committee of Shandong Provincial Hospital. Wild‐type AB strain zebrafish (Danio rerio), which is derived from A and B lines, and transgenic Tg (brn3c: GFP) were raised and maintained in fully automated zebrafish housing systems (ESEN, China; temperature 28 ± 0.5°C, pH 7.0, conductivity 500 μS) on a 14 h/10 h light/dark cycle and fed twice a day. Zebrafish embryos were obtained by natural spawning of adult zebrafish and transferred into 0.003% (w/v) 1‐phenyl‐2‐thiourea (PTU) before 24 hours to prevent pigmentation. At five days post‐fertilization (dpf), the larvae were obtained from hatched zebrafish embryos.

### 2.2 Drug preparation

CP was purchased from Sigma‐Aldrich and dissolved in DMSO to a concentration of 1 mmol/L. Neo and GM purchased from Beijing Suolaibao Technology Co., Ltd and dissolved in DMSO to a concentration of 2 mmol/L. Nar (Topscience, Beijing, China) was dissolved to a concentration of 1 mmol/L. The stock solution was then diluted to working concentration (Nar, 100‐400 μmol/L; CP, 150‐250 μmol/L; Neo and GM, 50‐150 μmol/L) in PTU. This dose was determined to be the most effective in pre‐experiment (unpublished data). All reagents were prepared immediately before each experiment.

### 2.3 Drug treatments

Zebrafish larvae at 5 dpf were treated with tested drugs in a 24‐well plate. We employed different protocols to investigate the otoprotective effects of Nar. To study Nar preventive and therapeutic effect, larvae were exposed to Nar for 3 hours and then co‐treated with Nar and ototoxic agent for the corresponding time (CP for 2 hours, GM for 3 hours, Neo for 1 hours). To study the effect of Nar on proliferation and apoptosis of HCs, larvae were co‐treated with Nar and ototoxic agent for a shorter time (CP for 1 hours, GM for 2 hours, Neo for 0.5 hours). The dose of ototoxicity we selected was found to be closest to the 50% inhibitory concentration (IC50) that caused HCs in the pre‐experiment.

To investigate the effect of Nar on HCs regeneration, larvae were treated with ototoxic drugs (CP for 3 hours, GM for 4 hours, Neo for 1 hours), the ototoxic drug action time window which showed in pre‐experiment that it could kill more than 99% of the lateral hair cells, and incubations were performed in the presence/absence of Nar 400 µmol/L before data collection. Control animals were exposed to vehicle (DMSO or PTU) and included in each experiment. Further experiments were performed after anaesthesia with 0.03% Tricaine (Sigma‐Aldrich, Shanghai, China) for 20 minutes.

### 2.4 Whole mount immunohistochemistry

The immunohistochemical experiment was carried out as mentioned earlier.^24^ The antibodies used in this study are as follows: anti‐Myosin VIIa antibody (1:200; DSHB) with Alexa Fluor 488 antibody (1:1,000; Invitrogen), anti‐BrdU antibody (1:500; Sigma‐Aldrich) with Alexa Fluor 594 secondary antibody (1:1,000; Invitrogen). Then, the larvae were incubated with DAPI (1:500 dilution; Thermo Fisher Scientific) for 20 min to label the nuclei.

### 2.5 TUNEL staining

For measuring the mitochondrial ROS production, HCs were labelled with MitoSOX probes (Thermo), according to the manufacturer's instructions. Larvae were incubated with 5 µmol/L MitoSOX Red Mitochondrial Superoxide Indicator for 10 minutes at 37°C. FM1‐43 (Molecular Probes, Invitrogen) was used to label functional HCs. FM1‐43 was prepared as a 5 μg/mL stock solution in ddH_2_O and diluted to a working concentration of 0.5 μg/mL in PTU. The solution was stocked in dark. Larvae were then incubated with FM1‐43 working solution for 1 minutes.

### 2.6 Living cells staining

To detect mitochondrial ROS production, HCs were labelled with MitoSOX probes (Thermo), according to the manufacturer's instructions. Larvae were incubated with 5 µmol/L MitoSOX Red Mitochondrial Superoxide Indicator for 10 minutes at 37°C; FM1‐43 (Molecular Probes, Invitrogen) was used to label functional HCs. FM1‐43 was made up of a stock solution of 5 μg/mL in ddH_2_O and diluted to a working concentration of 0.5 μg/mL FM1‐43 in PTU and kept away from light. Larvae were incubated with FM1‐43 for 1 minutes.

### 2.7 Microscopy

Zebrafish images were captured with a fluorescent microscope and confocal microscope (Nikon, Tokyo, Japan). Image data were processed with Imaris software (Imaris x64 9.2.1, Bitplane Inc, USA) for visualization. Fluorescence signal quantification was performed using Image‐Pro Plus software.

### 2.8 Neuromast morphological observation and cell count

In this study, we analysed five typical neuromasts that formed during different periods of primordium migration in the zebrafish lateral line: SO1 (supraorbital 1), O1 (otic 1), OC1 (occipital 1), D1 (dorsal trunk 1) and L1 (lateral 1). There are also three inner neuromasts: anterior, lateral and posterior crista. The positive cells per neuromast (NM) were counted under the fluorescence microscope in 10 fields at a magnification of 200×.

Neuromast morphology was ranked by using a scoring system as follows: 1 (the cell clusters are arranged neatly; the number and morphology of HCs are normal), 2 (the cell clusters are arranged neatly; a small number of HCs are lost, and the morphology is regular), 3 ( the cell clusters are normal; the number of HCs is reduced, and the hair cell bundles are disordered), 4 (the cell clusters are disorderly, a large number of HCs are lost and disordered) and 5 (the cell clusters are disordered, and the HCs are exhausted).

### 2.9 Antimicrobial susceptibility testing

Susceptibility testing was used to address whether Nar otoprotection interfered with the antibacterial efficacy of GM or Neo. The Escherichia coli strain was kindly provided by the microbiology laboratory, Shandong Provincial Hospital. In this experiment, Escherichia coli was first pre‐treated the sensitive tablets of gentamicin and neomycin (Suolaibao, Beijing, China) with Nar or vehicle. Then, drug sensitivity assays were performed according to standard procedures. Agar plates were imaged using a Leica MZ10 microscope, and the inhibitory area was calculated for each condition and compared with the corresponding control.

### 2.10 CCK‐8 assay

The cell counting kit‐8 assay was performed to investigate whether Nar oto‐protection affected the anti‐tumour activity of CP, and the effects on HEI‐OC1 cells which are an inner ear cell line.^25^ The HEI‐OC1 cells were friendly gifted by Dr Federico Kalinec from University of California, Los Angeles, which were maintained in DMEM medium with 10% FBS in 10% CO_2_ at 33°C.^26^ The Hela cells were preserved in our laboratory and maintained in DMEM medium with 10% FBS in 5% CO_2_ at 37°C. The cells were seeded into 96‐well plates and allowed to adhere for 24 h. Then, 10 μL of CP (10 μg/mL) with Nar (0‐200 μmol/L) or Nar alone was added to incubate with cells for 24 hours. Meanwhile, the cells incubated with vehicle were acted as a control group. Afterwards, 10 μL of CCK8 (Jiangsu Biyuntian Biotechnology Co., Ltd, China) was added into every well. After 1‐3 hours, absorbance at 450 nm was measured using a microplate reader (Molecular Devices). The cell viability ratio was calculated using the following formula: Cell viability ratio (%) = OD treated/OD control × 100.

### 2.11 RNA extraction and qPCR

Total RNA was extracted from whole larvae or HEI‐OC1 cells after corresponding treatment by using TRIzol Reagent (Invitrogen), according to the manufacturer's protocol. The RNA samples of 1 μg each were reversely transcribed into cDNA using a Kit (Takara, Japan) and then PCR‐amplified using the TaKaRa Taq Kit (Takara, Japan). Primer sets are described in Table S1.

### 2.12 Data analysis and statistics

For each treatment, data were taken from at least 10 samples and 3 experimental runs; for antimicrobial sensitivity tests, data were taken from 3 replicas and 3 experimental runs; and for CCK‐8 testing, data were taken from 3 copies and 3 experimental runs. qPCR quantification was conducted using the 2^−ΔΔCt^ method. All tests for significance were performed by Student's t test or Welch's t test. Differences were considered significant when *P* < 0.05.

## 3 RESULTS

### 3.1 Nar protects auditory HCs from CP‐induced cell death

At first, we tested the dose correlation of CP ototoxicity (Figure 1B). To evaluate the morphology of neuromast and the number of HCs following CP exposure, we used confocal immunofluorescence analysis. HCs were labelled with Myosin VIIa (green), and nuclei were labelled by DAPI. The result showed that all three concentrations of CP (150, 200 and 250 µmol/L) caused a significant loss of HCs in zebrafish lateral line. Besides, this ototoxicity of CP did not differ in a dose‐dependent manner (Figure 1B, D).

To assess whether Nar can protect zebrafish HCs from CP‐induced HCs injury, zebrafish larvae (5 dpf) were pre‐treated with 0 µmol/L to 400 µmol/L of Nar for 3 hours and then co‐treated with Nar and CP (200 µmol/L) for 2 hours (Figure 1B‐C). The results showed that pre‐treatment and co‐treated with Nar could effectively increase the number of surviving hair cells for CP treatment (Figure 1B). Furthermore, the obtained results were well correlated with the dose concentration, and the optimal concentration for Nar is 400 μmol/L (Figure 1E).

Accordingly, we sought to determine whether Nar also plays a role in the morphology of neuromast. Figure 1F showed that scores for neuromast morphology were also improved when CP‐exposed larvae were treated with different concentrations of Nar. Collectively, these results suggest Nar can protect HCs from the cytotoxicity of CP.

### 3.2 Nar protects HCs against GM‐induced toxicity

**FIGURE 2:**
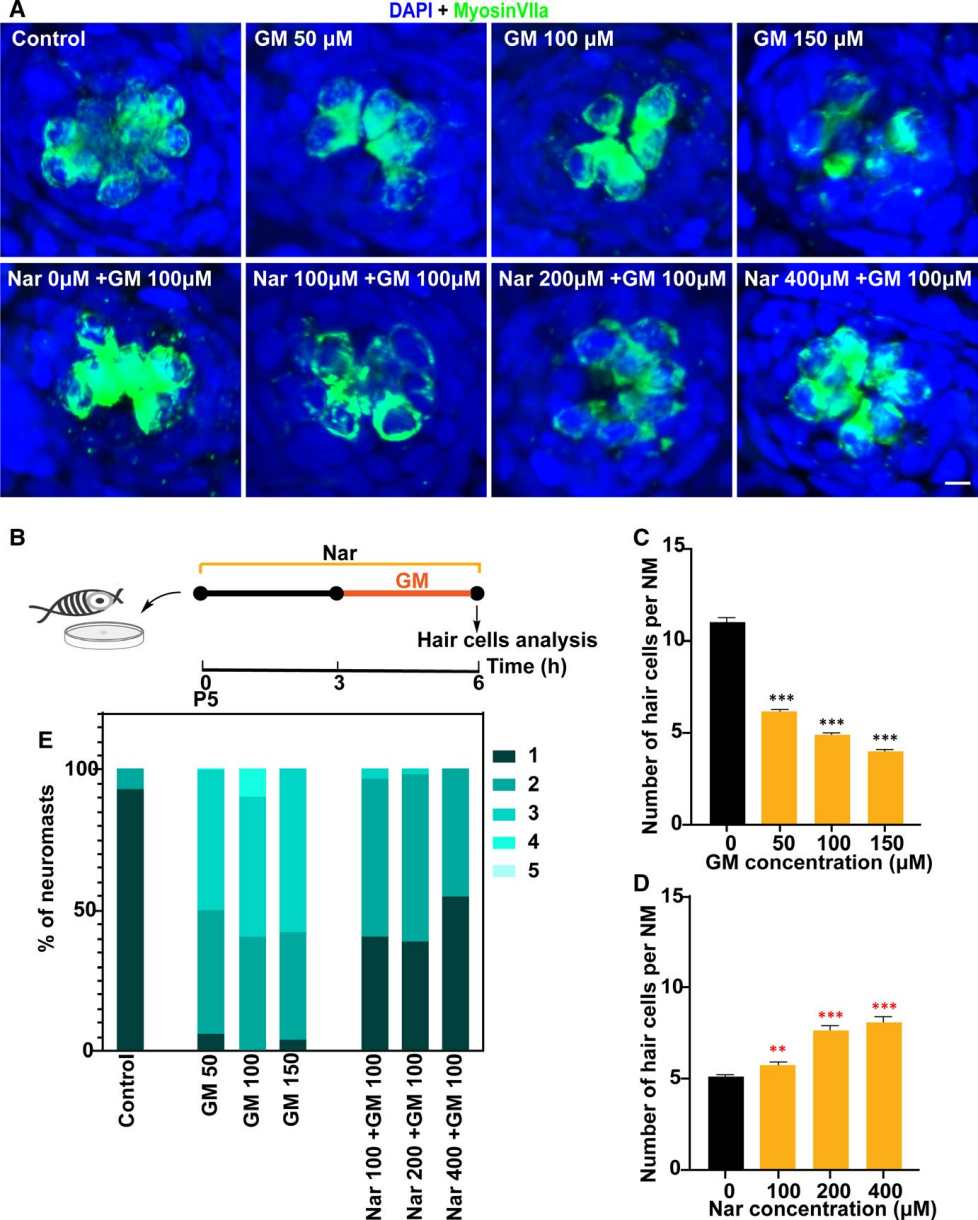
Nar protects against gentamicin ototoxicity. (A) Animals were fixed and immunostained for MyosinVIIa (green) and DAPI. 5 dpf zebrafish were incubated with 0 μmol/L to 150 μmol/L GM for 3 h or pre‐treated with 0 µmol/L to 400 µmol/L of Nar for 3 h and then co‐treated with Nar and GM (100 µmol/L) for 3 h. (B) Diagram of the assay for A. (C and D) Quantification of the number of hair cells (MyosinVIIa^+^) per neuromast after the different treatments represented as mean ± SEM (n = 10). Control animals were exposed to vehicle alone (DMSO). **P < 0.01, ***P < 0.001. Black asterisks compared to DMSO‐treated animals. Red asterisks compared versus the corresponding GM concentration. (E) Scores for neuromast morphology of each group (see Materials and Methods). Scale bar equals 5μm

We then applied the same method to investigate the influence of Nar on the ototoxicity effect of aminoglycosides. Firstly, we tested the cytotoxicity of GM on HCs within 3 hours (Figure 2A‐B). It was seen that the number of surviving HCs was decreased from about 7 to 3 per NM decreases with increasing doses of GM (50‐150 M) accordingly (Figure 2C). Then, we examined the ability of Nar to antagonize GM ototoxicity. In this experiment, 5‐dpf zebrafish were pre‐treated with 0 µmol/L to 400 µmol/L of Nar for 3 hours and then co‐treated with Nar and GM (100 µM) for 3 hours (Figure 2A‐B). The results showed that Nar treatment significantly increased HCs survival in GM‐exposed larvae (Figure 2D).

Similarly, we found that GM also interfered with the morphology of neuromast, resulting in lower scores for neuromast morphology. By contrast, Nar treatment reversed this reduction to a certain extent (Figure 2E). And the effect of regulation is a dose‐dependent manner.

### 3.3 Nar protects HCs against Neo‐induced acute toxicity

**FIGURE 3:**
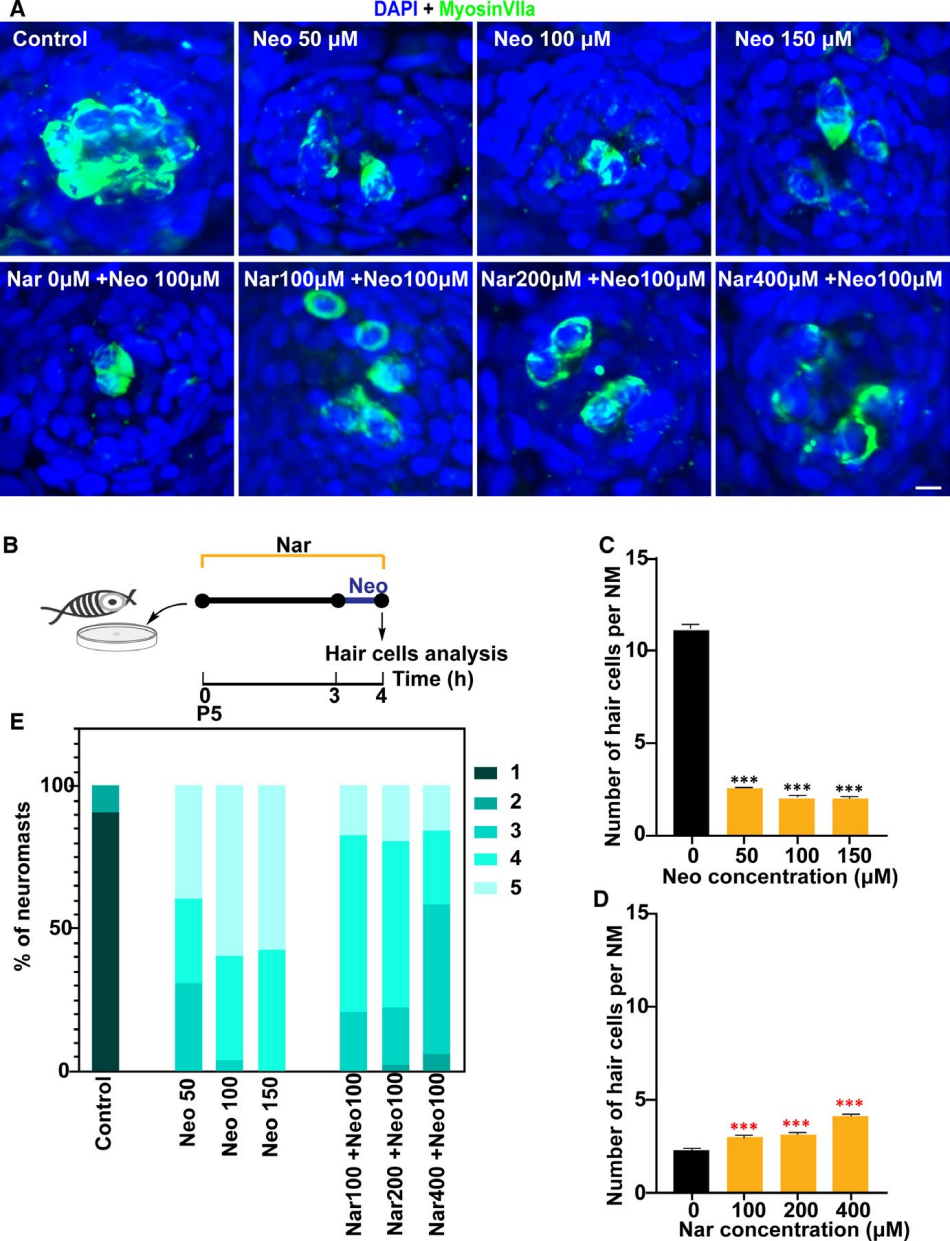
Nar protects against neomycin ototoxicity. (A) Animals were fixed and immunostained for MyosinVIIa (green), and DAPI. 5 dpf zebrafish were incubated with 0 μmol/L to 150 μmol/L Neo for 1 h, or pre‐treated with 0 µmol/L to 400 µmol/L of Nar for 3 h and then co‐treated with Nar and Neo (100 µmol/L) for 3 h. (B) Diagram of the assay for A. (C and D) Quantification of the number of hair cells (MyosinVIIa^+^) per neuromast after the different treatments represented as mean ± SEM (n = 10). Control animals were exposed to vehicle alone (DMSO). ***P < 0.001. Black asterisks compared to DMSO‐treated animals. Red asterisks compared versus the corresponding Neo concentration. (E) Scares for neuromast morphology of each group(see Materials and Methods). Scale bar equals 5 μm

Aminoglycosides were always used in combination.^27^ Therefore, we further tested whether Nar had an otoprotection effect for neomycin, another aminoglycoside drug. Firstly, the results showed that neomycin had a strong killing effect on HCs, leading to an 80% loss when larvae were treated with 50‐150 μmol/L of drug for only 1 hour (Figure 3A‐C). Again, we observed that co‐treatment of Nar increased the survival of neomycin‐exposed HCs in a dose‐dependent manner (Figure 3A,D). Of note, we found that Neo treatment severely damaged the morphology of neuromast, and the neuromast is almost destroyed by the end of the experiment. The results showed that this trend was reversed by Nar to some extent (Figure 3E). Taken together, these results suggested that Nar could protect HCs against the cytotoxicity of aminoglycosides.

Besides this, we also observed the inner ear HCs of transgenic Tg (brn3c: GFP) zebrafish treated with media or ototoxic drugs. Figure S1A showed that the inner ear HCs (anterior, lateral and posterior crista) perpendicular to each other was still in a state of the intact structure and towering cilia when larvae were treated with CP (200 μmol/L), GM (100 μmol/L) or Neo (100 μmol/L), but the HCs in the lateral line were seriously damaged. We also compared the number of HCs in different positions of the inner ear of each group. The results, as shown in Figure S1B, indicated that the total number of HCs in each group with different treated was not statistically different, and the number of HCs in different positions was different. Further statistical tests revealed that both CP and aminoglycosides did not decrease the morphological fraction of the inner auricular neuromast of zebrafish (Figure S1C).

### 3.4 Nar promotes supporting cell proliferation in neuromasts

**FIGURE 4:**
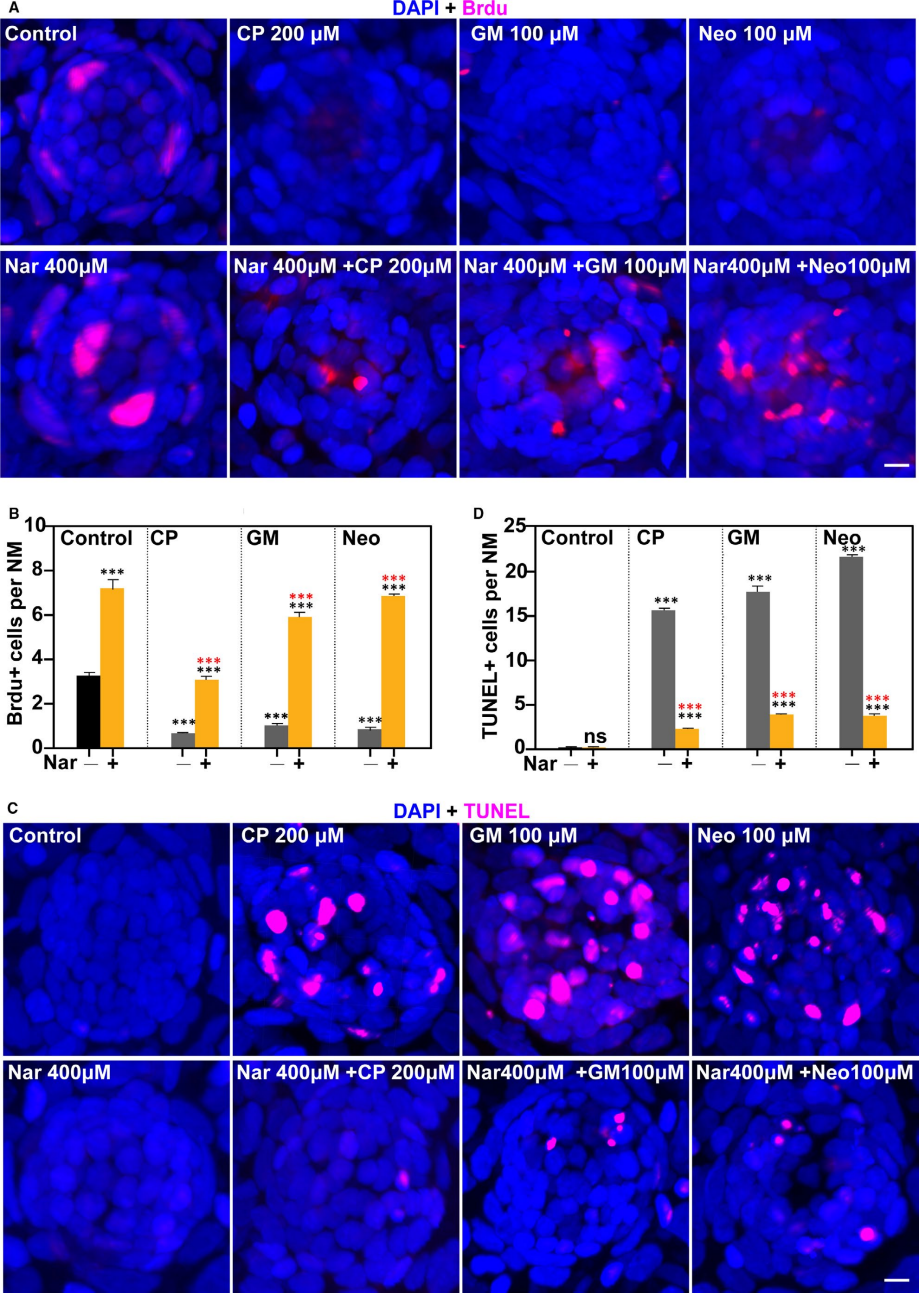
Nar promotes cell proliferation and protects against ototoxin‐induced cell death in neuromasts. (A) Proliferation assays were performed in 5 dpf zebrafish in the presence/absence of Nar (400 µmol/L) and the corresponding ototoxin. Animals were fixed and immunostained for BrdU (pink) and DAPI. (B) The numbers of proliferated cells (BrdU^+^) per neuromast were calculated for each treatment and represented as mean ± SEM (n = 10). ***P < 0.001. Black asterisks compared versus corresponding control. Red asterisk compared versus the corresponding ototoxin‐only treatment. (C) TUNEL assay (red) was performed in zebrafish in the presence/absence of Nar (400 µmol/L) and the corresponding ototoxin. (D) The numbers of TUNEL‐positive cells per neuromast were calculated for each treatment and represented as mean ± SEM (n = 10). ***P < 0.001. Black asterisks compared versus corresponding control. Red asterisk compared versus the corresponding ototoxin‐only treatment. Scale bar equals 5 μm

To determine whether the oto‐protective function of Nar involved cell proliferation, we performed 5‐Bromo‐2‐deoxyUridine (BrdU, proliferation marker) immunofluorescence. 5‐dpf zebrafish were, respectively, incubated with different doses of ototoxic drugs, including CP (200 μmol/L) for 2 hours, GM (100 μmol/L) for 3 hours and Neo (100 μmol/L) for 1h. For oto‐protection group, zebrafish were pre‐treated with 400 µmol/L of Nar for 3 hours and then co‐treated with Nar and ototoxic drug for corresponding time. Immunofluorescence results indicated that BrdU‐positive cells were mostly located at the edge of neuromast (supporting cells are usually in this area) in control group and ototoxic drugs significantly reduced BrdU staining. It should be noted that a small number of BrdU‐positive cells appeared in the centre area of the damaged cell mass (The supporting cells at the bottom of HCs are usually in this area) (Figure 4A). By contrast, zebrafish co‐treated with Nar and ototoxic drugs showed a significantly increased number of BrdU‐positive cells, compared with ototoxic drugs treatment alone. And Nar treatment group had more proliferating (BrdU‐positive) cells than the control group (Figure 4A‐B). Overall, the data indicate that Nar can promote cell proliferation in neuromasts.

### 3.5 Nar prevents HCs death

To determine whether Nar prevents ototoxin‐induced HC death, we performed a Terminal deoxynucleotidyl transferase (TdT) dUTP Nick‐End labelling (TUNEL) assay. For this experiment, we applied the same drug treatment regimens as in BrdU assay (Figure 4C). The results showed that a large number of TUNEL‐positive cells appeared following 0.5‐2 hours of ototoxic drug treatment in neuromasts, and co‐treatment with Nar greatly reduced apoptotic cells. Besides, Nar treatment alone did not induce cell apoptosis in neuromasts (Figure 4D).

### 3.6 Nar reduced cilia damage and mitochondrial ROS accumulation caused by ototoxic drugs

**FIGURE 5:**
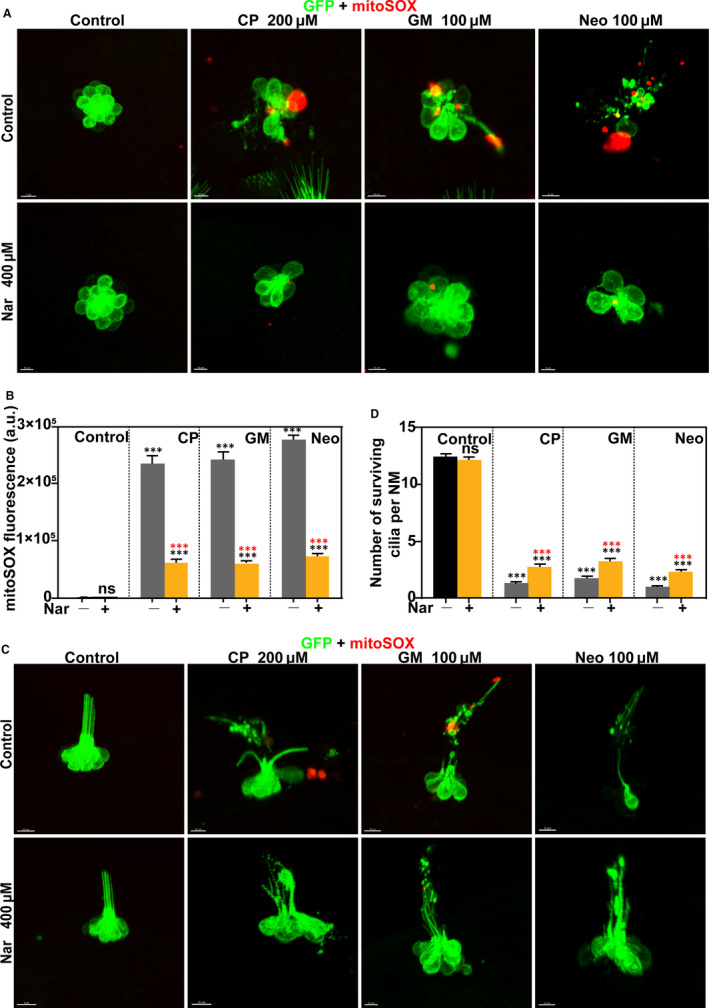
Protective effect of naringin against Mitochondrial oxidative stress and cilia damage. Zebrafish were treated with different concentrations of ototoxic drugs with or without Nar. Control animals were exposed to vehicle alone (DMSO). Still images of live brn3c: GFP hair cells (green) loaded with the live fluorescent indicator mitoSOX (red). (A) The top view of hair cell clusters to show the morphology of hair cells. (B) The mitoSOX fluorescent intensity per neuromast was calculated for each treatment and represented as mean ± SEM. ***P < 0.001. Black asterisks compared to DMSO‐treated animals. Red asterisks compared versus the corresponding ototoxin‐only treatment. (C) Side view of hair cell clusters to show hair cell cilia. (D) Quantification of the number of surviving cilia per neuromast after the different treatments represented as mean ± SEM. ***P < 0.001. Black asterisks compared to DMSO‐treated animals. Red asterisks compared versus the corresponding ototoxin‐only treatment

Evidence has shown that the production of mitochondrial ROS has been strongly linked to aminoglycosides or CP‐induced ototoxicity.^28^ We, therefore, wondered whether Nar played a role in this process. To attain this objective, we evaluated mitochondrial ROS levels with the live fluorescent indicator mitoSOX. Also, we established a Tg (brn3c: GFP) transgenic zebrafish line, which expressed a membrane‐bound GFP in hair cells. (Figure 5A, C).

Animals were incubated with the ototoxin in the presence/absence of 400 μmol/L of Nar. The stained cells were then analysed using a confocal microscope. As shown in Figure 5B, Nar treatment reduced CP and aminoglycosides‐induced mitochondrial ROS production and accumulation.

Furthermore, we observed that stereociliary morphogenesis is drastically impaired after being treated with ototoxic drugs (Figure 5C). The normal stereocilia are dense and straight, while the damaged ones are bent and broken. In the following, we presented a statistic comparison of the number of surviving cilia with each group. The results showed that Nar could alleviate the cilia deformity caused by CP and aminoglycosides (Figure 5D).

### 3.7 Nar promotes hair cell regeneration and functional recovery

**FIGURE 6:**
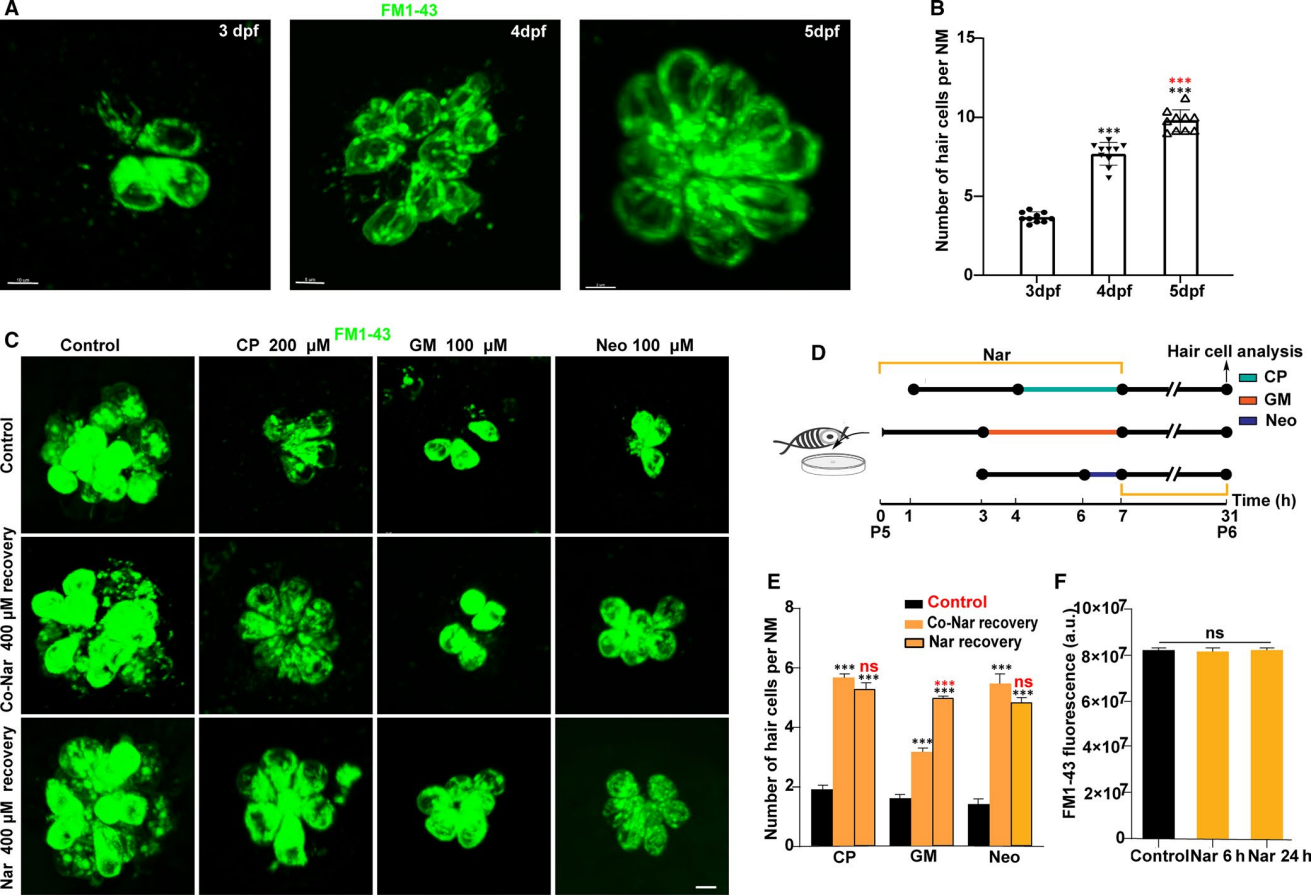
Naringin promotes the regeneration of functional hair cells. A, Still imaging of hair cells of zebrafish larvae 3, 4 and 5 d after FM1‐43 labelled (green) fertilization. B, Scatter plot of FM1‐43 labelled hair cell count at each time‐point (***P < 0.001; mean ± SEM; n = 10). Black asterisks correspond to 3 dpf animals, red asterisks correspond to 4 dpf animals. Hair cells with mechanotransduction channel activity (FM1‐43^+^, green) are defined as functional and mature hair cells. (C) Still imaging of hair cells after different treatment: 5 dpf Wild‐type larvae were incubated with vehicle alone for 24 h after being treated with vehicle or serial dilutions of Ototoxic drugs (*Control group*), or with Nar 400 µmol/L prevention and co‐incubation treatment (*Co‐Nar recovery group*); larvae were treated by ototoxic drugs and then incubated with Nar for 24 h (*Nar recovery group*); 5 dpf zebrafish were incubated with Nar for 6 h or 24 h as the corresponding control. Scale bar equals 5 µm. (D) Diagram of the assay for C. (E) The FM1‐43 fluorescent intensity per neuromast was calculated for each treatment and represented as mean ± SEM. No significant differences were observed between control and treated animals (unpaired Student's *t* test). (F) Quantification of the number of functional hair cells per neuromast after the different treatments represented as mean ± SEM. ***P < 0.001. Black asterisks compared versus control. Red asterisks compared versus the corresponding Nar concentration

Evidence shows that hair cell loss in zebrafish can be fully restored through tissue regeneration, which involves stem cell proliferation, differentiation and cell death.^29^ Therefore, we hypothesized that Nar would promote HCs' mass regeneration following ototoxic damages. In addition, to understand the potential of Nar in promoting the functional recovery of damaged HCs cluster, we tested hair cell mechanotransduction by using FM1‐43, a fluorescent dye that passes through MET channels (Figure 6A). In Figure 6B, we can see a very obvious upward trend of FM1‐43 labelled HCs as the larvae grow. An implication of this is the possibility that FM1‐43 can be used as a marker of hair cell maturity.

Here, we took three different strategies (Figure 6C‐D). Group 1 (Control group):5‐dpf Wild‐type larvae were incubated with vehicle alone for 24 hours after being treated with vehicle or serial dilutions of Ototoxic drugs (CP 200 μmol/L for 3 hours, GM 100 μmol/L for 4 hours, Neo 100 μmol/L for 1 hours); Group 2 (Co‐Nar recovery group): Larvae were pre‐incubated with Nar (400 μmol/L) for 3 hours, followed co‐incubation with Nar and with ototoxic drugs for a certain period, and then incubated with vehicle alone for 24 hours; Group 3 ( Nar recovery group):5‐dpf Wild‐type larvae were incubated with Nar for 24 hours after being treated with vehicle or serial dilutions of ototoxic drugs.

The results showed that treatment with Nar significantly increased the number of HCs in the recovery period after injury. Then, we analysed the statistical difference between the effect of co‐Nar recovery group and Nar recovery group: The difference between the two methods was not statistically significant treated by CP and Neo (*P* > 0.05), and Nar recovery group treated with GM is more effective than co‐Nar recovery group (*P* < 0.001) (Figure 6E).

On the other hand, it has been reported that the way CP and aminoglycosides enter HCs is related to mechanical conduction channels. Therefore, we wanted to investigate whether Nar could protect HCs by shutting down mechanical pathways. By analysing the fluorescence intensity of FM1‐43, mechanotransduction activity of HCs could be measured and compared. The results showed that treatment with Nar did not affect hair cell mechanotransduction activity at any time‐point (Figure 6F). These results demonstrate that Nar promotes the regeneration of functional HCs after injury.

### 3.8 Nar does not affect aminoglycosides' efficacy

To investigate whether Nar interfered with the efficacy of antibiotics and chemotherapy drugs, we performed the disc susceptibility tests of Escherichia coli. Escherichia coli were exposed to GM and Neo alone or in the presence of 400 µmol/L of Nar and the inhibitory area calculated after overnight incubation. The results showed that Nar did not affect the antibacterial activities of aminoglycosides (Figure S2A‐B).

### 3.9 Nar could enhance the efficacy of CP

Next, we performed a cytotoxic assay against HeLa cells with CP and Nar. The results showed that naringin promoted the killing effect of cisplatin on tumour cells (Figure S2C). To provide further evidence for Nar did not appear to be toxic for HCs in vitro, given that cytotoxicity of Nar against tumour cells, we did CCK8 assay of the proliferation ability of the indicated HEI‐OC1 cells with Nar treatment. The results show that Nar can enhance the proliferation of HEI‐OC1 cells (Figure S2D). Our findings suggest that Nar alleviated the ototoxicity of CP and aminoglycosides without compromising their therapeutic efficiencies.

### 3.10 Effect of Nar on regulating apoptotic‐ and pyroptosis‐related genes

**FIGURE 7:**
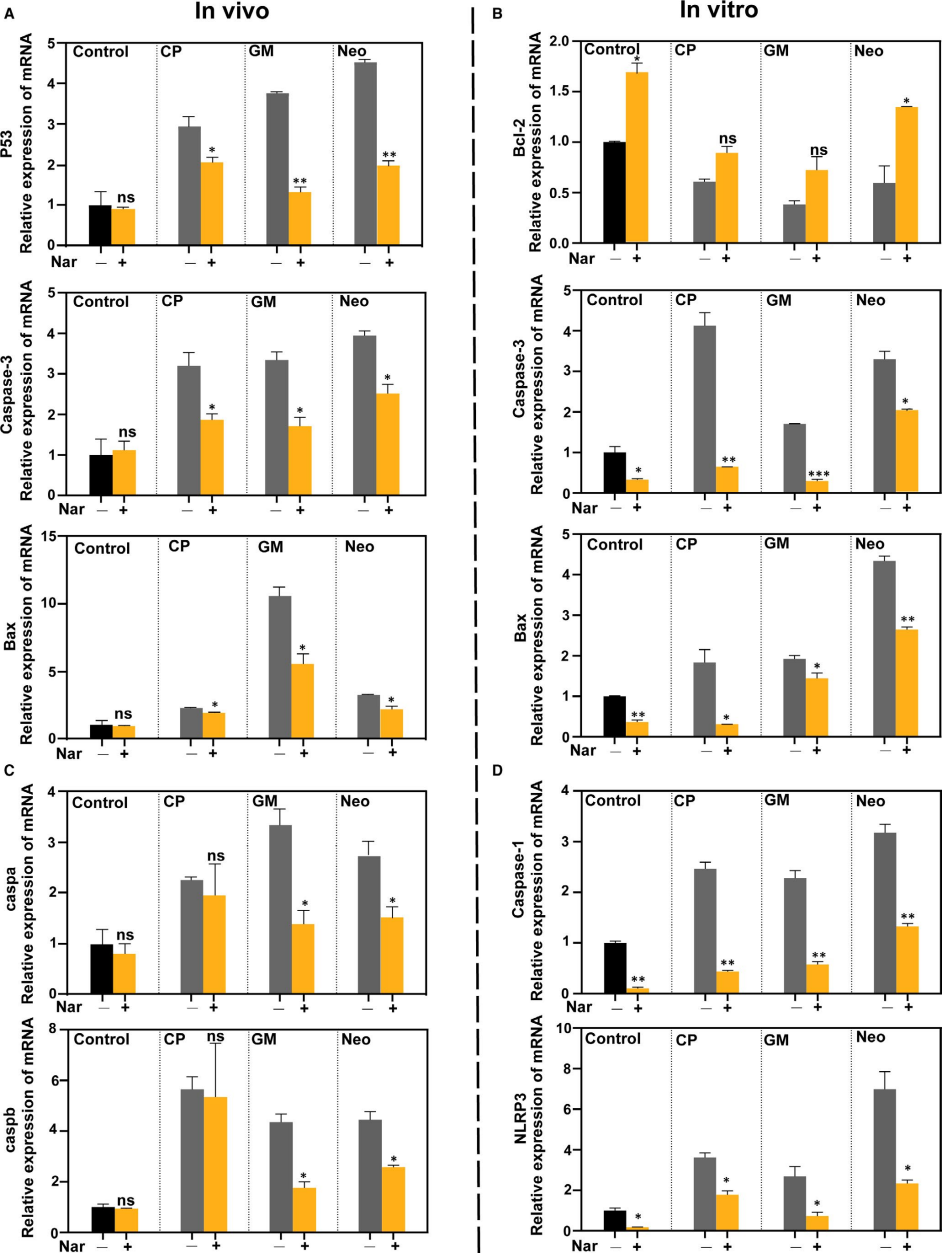
Naringin attenuates the activation of the apoptotic‐ and pyroptosis‐related pathway in vitro and in vivo. (A and C) Real‐time PCR analysis of the mRNA levels of apoptotic (*P53, Bax, Caspase‐3*) and pyroptosis (*caspb, caspa*) related genes in zebrafish larvae treated with the presence/absence of Nar (400 µmol/L) and the corresponding ototoxin (CP 200 µmol/L, GM 100 µmol/L, Neo 100 µmol/L). Data are expressed as the mean ± SEM. ***P < 0.001, **P < 0.01, *P < 0.05. Black asterisks compared versus corresponding control. (B and D) Real‐time PCR analysis of the mRNA levels of apoptotic‐ (*Bcl‐2, Bax, Caspase‐3*) and pyroptosis‐ (*Caspase‐1, NLRP3*) related genes in HEI‐OC1 cells treated with the presence/absence of Nar (100 µmol/L) and the corresponding ototoxin (CP 10 µg/mL, GM 50 µmol/L, Neo 50 µmol/L). Data are expressed as mean ± SEM. ***P < 0.001, **P < 0.01, *P < 0.05. Black asterisks compared versus corresponding control

To investigate the mechanisms by which Nar prevented HCs death induced by CP and aminoglycosides, we quantified the mRNA levels of apoptotic‐ (*P53* or *Bcl‐2*, *Bax* and *Caspase‐3*) and pyroptosis‐ (*caspb* and *caspa* or *Caspase‐1* and *NLRP3*) related genes via qPCR in vivo and vitro study. The results showed that Nar alone did not affect the mRNA levels of apoptotic‐ and pyroptosis‐related genes, compared with control group in vivo study (Figure 7A,C).

By contrast, Nar significantly reduced the CP‐ and aminoglycoside‐induced up‐regulation of *P53*, *Bax* and *Caspase‐3* when compared with the ototoxic drug‐alone group (Figure 7A). And Nar also down‐regulated the expression of pyroptosis‐related genes compared to the aminoglycosides alone, but the CP group showed no such changes (Figure 7C).

Meanwhile, to varying degrees, Nar decreased the activity of the apoptotic‐ and pyroptosis‐related pathway in vitro study as shown in Figure 7B,D. These results demonstrated that Nar suppressed the expression of apoptotic‐ and pyroptosis‐related genes to protects HCs.

## 4 DISCUSSION

Because of the similarity of pharmacological specificity to mammals/birds, zebrafish model of human disease is particularly suitable for pharmacological testing in a convenient way.^30^ But if we want to translate laboratory research results into the clinical application, we must recognize the difference between zebrafish models and mammals. In humans and mice, CP and aminoglycosides must pass through a series of tissues to invade HCs. First, they need to enter the inner ear tissue through blood‐labyrinth barrier (BLB), then from endolymph to the parietal membrane of HCs, and finally, enter HCs through channels such as MET.^31^ By contrast, zebrafish could uptake CP and aminoglycosides independently and continuously from the solution through the skin and cavity (mouth).^32^ Particularly, HCs located on the body surface of zebrafish can be directly exposed to ototoxic drugs in a short period. In this study, we found that the number of zebrafish lateral HCs exposed to CP and aminoglycosides decreased rapidly within 3 hours, which proved an acute toxic reaction. On the other hand, the transparent character of zebrafish larvae allows us to directly observe HCs of zebrafish lateral line by using a transgenic zebrafish Tg (brn3c, GFP). We found that neither CP nor aminoglycosides caused damage to the HCs of the inner ear during the experimental time, which suggests that we need to use different ways of administration to study the HCs of the inner ear of zebrafish, such as arteriovenous injection, oral administration or direct microinjection into the ear capsule.

In our experiment, the preventive and co‐treatment of Nar significantly reduced the ototoxicity of CP and aminoglycosides on HCs. In this process, the hair cells are directly exposed to the drug. While we should also be soberly aware that this mode of administration is equivalent to injecting drugs directly into the inner ear of mice or humans without going through the digestive system and blood circulation. Unknown questions include whether Nar (molecular weight 580.53) can pass through the blood maze barrier, whether it will affect the environmental homeostasis of endolymph, and whether oral and intravenous administration can also be effective. On the other hand, there is evidence that Nar has no acute and subchronic toxicology in humans, which shows that Nar is a relatively safe therapeutic drug.

CP and aminoglycosides pass through the parietal membrane of HCs in different ways.^33^ CP uptake may occur through organic cation transporter 2 (OCT2) and copper transporter 1 (CTR1) transporters or through MET channel pores, for which the relative importance has not been established, most likely in the form of hydration. And CP also can pass through the cell membrane through free diffusion. On the other hand, aminoglycosides mainly pass through the cell membrane through the MET channels or endocytosis by the apical membrane (eg transient receptor potential V1[TRPV1] and TRPV4). It should be noted that zebrafish HCs do not express CTR1 or OCT2.^14^ This is probably because the cisplatin enrichment of hair cells in zebrafish is far less than that of amino glucoside drugs, therefore, facilitating the ototoxic protection provided by NAR as we found that Nar showed improved protection of the HCs caused by CP than by GM and Neo.

Previous studies have shown that FM1‐43 passes the hair cells in zebrafish mainly through the calcium‐dependent MET channels.^34^ Therefore, we have applied the FM1‐43 staining label to detect the impact of Nar on MET channels. The results showed that Nar treatment did not affect mechanotransduction activity in the HCs. As there is evidence that naringenin (Naringenin is aglycone of Nar) affects the fluidity of cell membranes, the possibility of Nar alleviating ototoxicity by interfering with the MET channel of ototoxic drugs into HCs cannot be ruled out.^23^, ^35^ However, the hypotheses need to be confirmed by more experiments, such as detecting the concentration of Nar in HCs and identifying molecular targets.

The disordered arrangement of HCs in inner ear causes hearing damage, so we also investigated the morphological changes of zebrafish neuromast. The neuromast is mainly composed of supporting cells around and at the bottom, hair cell clusters at the centre and top, and outer mantle cells arranged in a rose pattern.^36^ We found that both CP and aminoglycosides caused a decrease of neuromast morphological fraction, and this damage could be partly reversed by Nar. This suggests that, besides HCs, ototoxic drugs also induce damage to supporting cells, which is consistent with previous research. In the subsequent TUNEL assay, we also found that the apoptotic signals caused by CP and aminoglycosides were distributed in the whole neuromast and similarly Nar significantly reduced the apoptotic signals of the whole neuromast. These results suggest that Nar can resist apoptosis induced by ototoxic drugs in whole neuromast, not only HCs.

Previous studies have pointed out that Nar has the potential to promote cell proliferation and differentiation.^37^ Here we found that there were still proliferative cells in the lateral neuromast of 5‐day‐old zebrafish larvae, mainly in the periphery of the neuromast. After treatment with CP and aminoglycosides, these cells decreased, while a small number of proliferation signals appeared in the central area of the neuromast. We speculate that this may be due to the spontaneous repair mechanism of HCs. It was worth noting that, after Nar treatment, the number of proliferative cells in neuromast was greatly increased. These results suggest that the protective effect of Nar against ototoxic drugs was, at least partly, through promoting the proliferation of neuromast cells. Given this result, we then tested whether Nar could promote the regeneration of zebrafish HCs.

Previous studies have shown that adult mammals including human cochlear HCs lack the ability of spontaneous regeneration. In recent years, it is possible to regenerate adult mouse inner ear HCs through the induction and differentiation of exogenous genes. Therefore, promoting hair cell regeneration after injury may be another way to solve the problem of drug ototoxicity. The study on the mechanism of inducing zebrafish hair cell regeneration has become a research hotspot.^38^ In this study, we focused not only on the recovery of HCs amount after injury but also on the changes in their function. MET channel is the pathway of acoustoelectric conversion, so its status represents the normal function of HCs. We found that FM1‐43 staining was continuously increased as the embryo matured (3‐6 dpf) which suggested that FM1‐43 could represent the differentiation degree of HCs. Besides, compared with CP or aminoglycoside alone, co‐treatment with Nar significantly increased the number of FM1‐43‐labelled HCs. Therefore, we think that Nar may help the recovery of HCs after injury by promoting the proliferation and differentiation of HCs.

Another potential mechanism of the ototoxicity of CP and aminoglycosides is an intracellular accumulation of reactive oxygen species.^17^ Therefore, we studied the effect of Nar on mitochondrial reactive oxygen species caused by ototoxicity. The evidence shows that Nar treatment significantly reduced the level of reactive oxygen species in mitochondria, which is consistent with our expectations (the antioxidant effect of Nar has been confirmed by previous studies^19^). Also, with the help of transgenic zebrafish, we observed that ototoxic drugs caused cilia bending (more common in the CP group) and breakage (common in aminoglycoside group) before HCs apoptosis. Although this trend can be alleviated to some extent by Nar, the mechanism needs to be further investigated.

In addition, an ideal anti‐ototoxicity measure should not weaken the therapeutic efficacy of drugs. For this reason, we investigated the effects of Nar on the antibacterial activity of aminoglycosides and anti‐tumour activity of CP. The results showed that Nar did not affect the bactericidal effect of antibiotics, and further enhance CP curative effect. This is consistent with previous studies that have assessed the impact of Nar on tumour cells.^39^ The difference shown here may be due to a difference in signalling pathways activated in the two different cell types. This reveals a high utility of Nar.

Finally, in order to explore the molecular mechanism of Nar in the anti‐hair cell death, we measured the RNA levels of genes associated with apoptosis and pyroptosis in vivo and in vitro, respectively. The results showed that it could reduce the activation of apoptosis or pyroptosis‐related pathways. It is worth noting that it plays an important role in the anti‐pyroptosis, and the relevant mechanisms need to be further explored.

In conclusion, this study demonstrated for the first time the oto‐protective effect of Nar against the ototoxicity of CP and aminoglycosides and explained the possible mechanisms. We provided evidence that Nar could support the survival of functional HCs in terms of anti‐apoptosis, antioxidation, proliferation and differentiation and emphasize its potential in clinical application.

## CONFLICT OF INTEREST

The authors declare that they have no conflict of interest.

## AUTHOR CONTRIBUTION

**Ming Li:** Data curation (equal); Methodology (equal); Writing‐original draft (equal); Writing‐review & editing (equal). **Jingwen Liu:** Methodology (equal). **Dong Liu:** Project administration (equal). **Xuchu Duan:** Methodology (equal). **Qingchen Zhang:** Methodology (equal). **Dawei Wang:** Methodology (equal). **Qingyin Zheng:** Writing‐review & editing (equal). **Xiaohui Bai:** Data curation (equal); Writing‐review & editing (equal). **Zhiming Lu:** Data curation (equal); Writing‐review & editing (equal).

## Supporting information

Fig S1

Fig S2

Table S1

## Data Availability

The data that support the findings of this study are available from the corresponding authors upon reasonable request.
