# Supplementary figures and images for: Naringin attenuates cisplatin‐ and aminoglycoside‐induced hair cell injury in the zebrafish lateral line via multiple pathways
Source: J Cell Mol Med. 2020 Dec 3;25(2):975–89. doi: 10.1111/jcmm.16158 (PMC7812295; doi:10.1111/jcmm.16158)

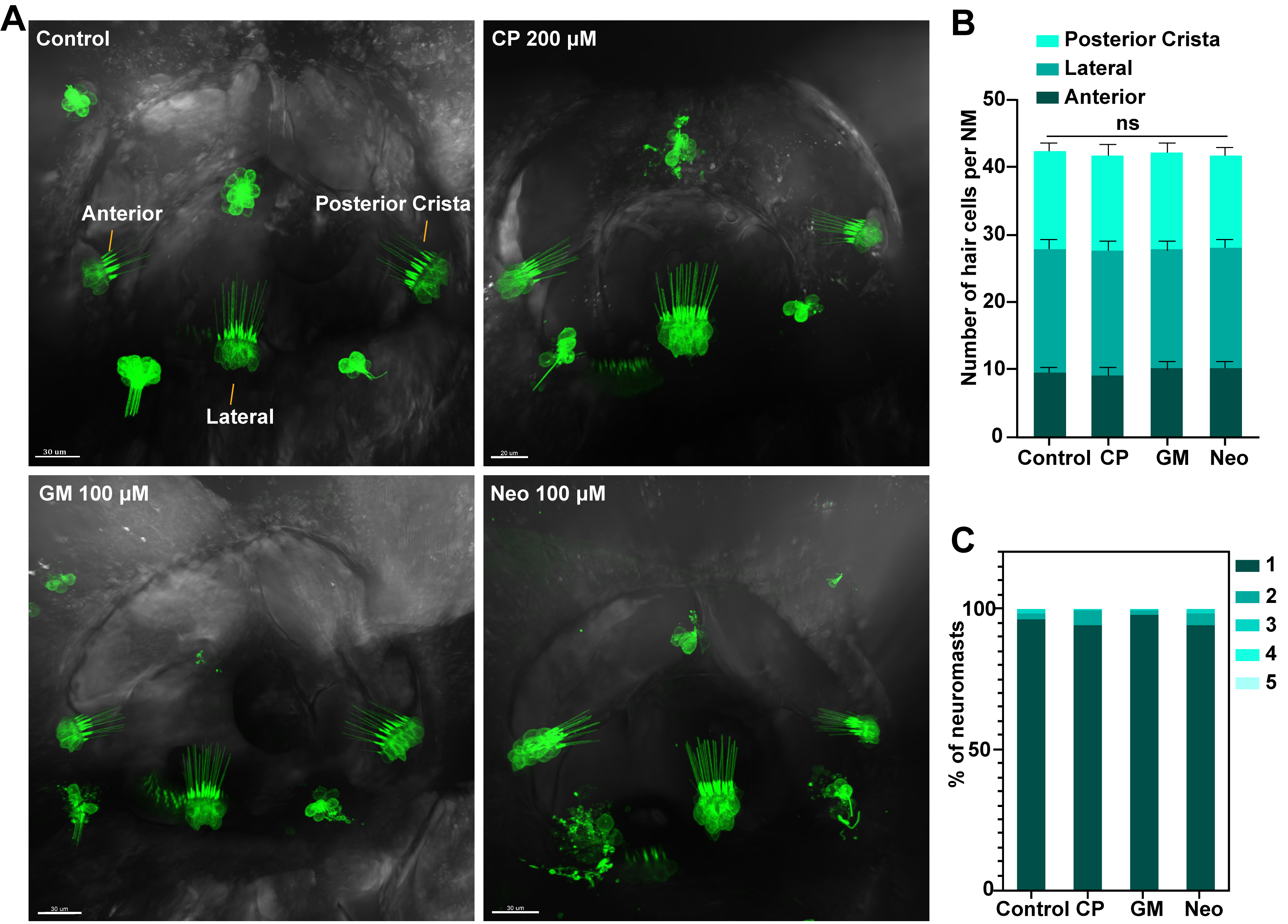

Supplement: Supplementary file 1 — Fig S1 [file JCMM-25-975-s001.png]

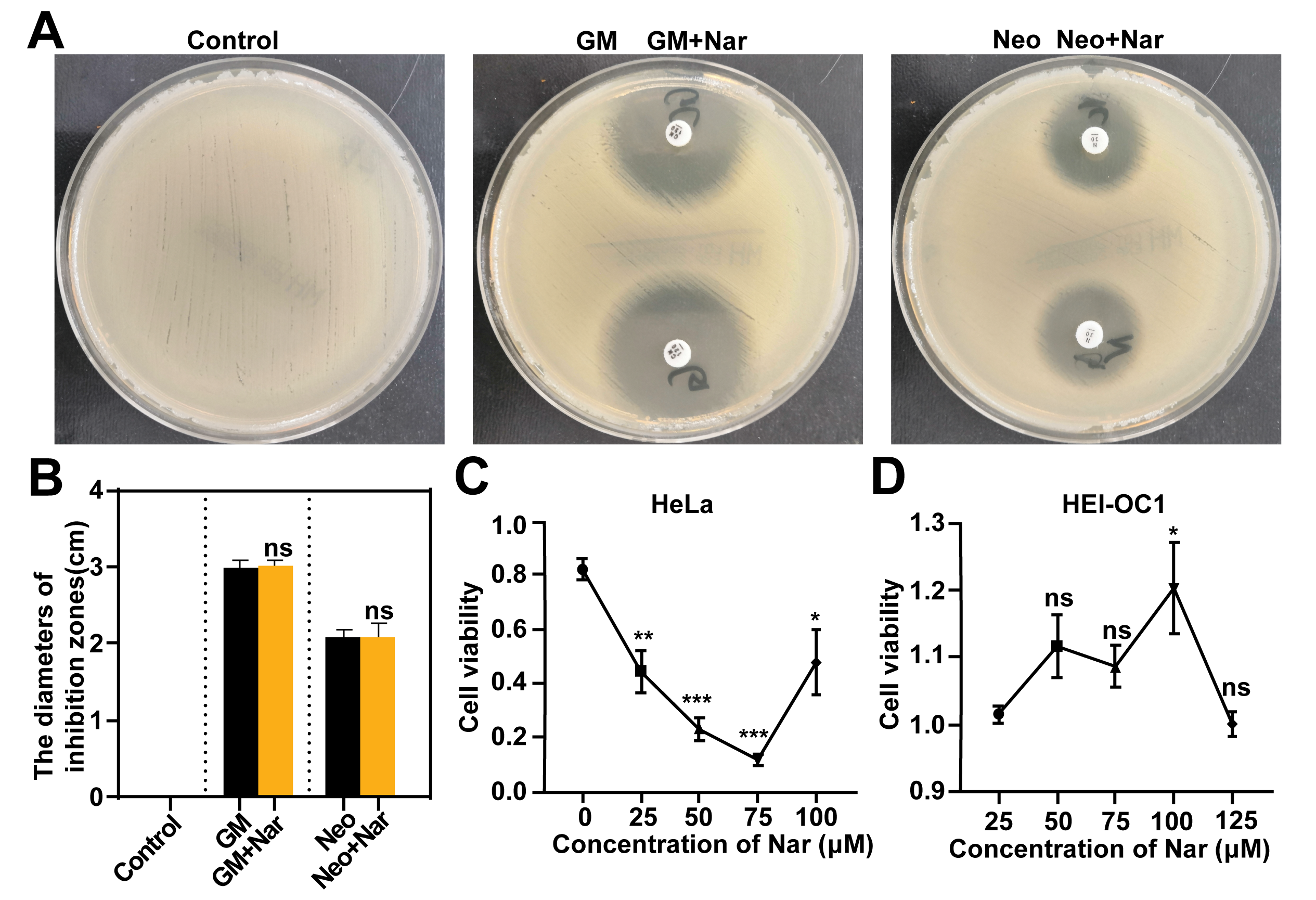

Supplement: Supplementary file 2 — Fig S2 [file JCMM-25-975-s002.png]
