# Supplementary material for: Naringin attenuates cisplatin‐ and aminoglycoside‐induced hair cell injury in the zebrafish lateral line via multiple pathways
Source: J Cell Mol Med. 2020 Dec 3;25(2):975–89. doi: 10.1111/jcmm.16158 (PMC7812295; doi:10.1111/jcmm.16158)
Supplement: Supplementary file 3 — Table S1 [file JCMM-25-975-s003.doc]

**TABLE S1 The primers sequences of relevant genes designed for qPCR.**

| Primer name | Species | Direction | Primer sequence |
| --- | --- | --- | --- |
| P53-F | zebrafish | 5′-3′ | CCACCATGAGAGAACACCTGAT |
| P53-R | zebrafish | 5′-3′ | GCTGAGGAGCTTCATAGAGAACC |
| Bax-F | zebrafish | 5′-3′ | GGAGGCGATACGGGCAGT |
| Bax-R | zebrafish | 5′-3′ | TTGCGAATCACCAATGCTGTG |
| Caspase-3-F | zebrafish | 5′-3′ | TTACTCCACCGCACCAGGAT |
| Caspase-3-R | zebrafish | 5′-3′ | TAAGAGCATGTCGCACAGCG |
| caspa-F | zebrafish | 5′-3′ | CGGTGAGCCTGATGAGCCAATG |
| caspa-R | zebrafish | 5′-3′ | TTCCATTCTGCACATGCCGGTAAG |
| caspb-F | zebrafish | 5′-3′ | GTGAAACCAGGCGTCGAACCC |
| caspa-R | zebrafish | 5′-3′ | CTGTACTGCTGAACCATGCTGTCC |
| b-actin-F | zebrafish | 5′-3′ | TACAGCTTCACCACCACAGC |
| b-actin-R | zebrafish | 5′-3′ | AAGGAAGGCTGGAAGAGAGC |
| Bax-F | mouse | 5′-3′ | TCAGGATGCGTCCACCAAGAAG |
| Bax-R | mouse | 5′-3′ | TGTGTCCACGGCGGCAATCATC |
| Caspase-3-F | mouse | 5′-3′ | GGAAGCGAATCAATGGACTCTGG |
| Caspase-3-R | mouse | 5′-3′ | GCATCGACATCTGTACCAGACC |
| Bcl-2-F | mouse | 5′-3′ | ATCGCCCTGTGGATGACTGAGT |
| Bcl-2-R | mouse | 5′-3′ | GCCAGGAGAAATCAAACAGAGGC |
| Caspase-1-F | mouse | 5′-3′ | GCCTGTTCCTGTGATGTGGAG |
| Caspase-1-R | mouse | 5′-3′ | TGCCCACAGACATTCATACAGTTTC |
| NLRP3-F | mouse | 5′-3′ | GTTTTCATTCCTGCACTGCCAGTG |
| NLRP3-R | mouse | 5′-3′ | CAAAAACCCTTCTGTTTACTCACTC |
| GAPDH-F | mouse | 5′-3′ | TTCCTACCCCCAATGTATCCG |
| GAPDH-R | mouse | 5′-3′ | CATGAGGTCCACCACCCTGTT |
